# Modelling the Effects of Electrical Coupling between Unmyelinated Axons of Brainstem Neurons Controlling Rhythmic Activity

**DOI:** 10.1371/journal.pcbi.1004240

**Published:** 2015-05-08

**Authors:** Michael J. Hull, Stephen R. Soffe, David J. Willshaw, Alan Roberts

**Affiliations:** 1 Institute for Adaptive and Neural Computation, University of Edinburgh, Edinburgh, United Kingdom; 2 School of Biological Sciences, University of Bristol, Bristol, United Kingdom; Northeastern University, UNITED STATES

## Abstract

Gap junctions between fine unmyelinated axons can electrically couple groups of brain neurons to synchronise ﬁring and contribute to rhythmic activity. To explore the distribution and significance of electrical coupling, we modelled a well analysed, small population of brainstem neurons which drive swimming in young frog tadpoles. A passive network of 30 multicompartmental neurons with unmyelinated axons was used to infer that: axon-axon gap junctions close to the soma gave the best match to experimentally measured coupling coefﬁcients; axon diameter had a strong inﬂuence on coupling; most neurons were coupled indirectly via the axons of other neurons. When active channels were added, gap junctions could make action potential propagation along the thin axons unreliable. Increased sodium and decreased potassium channel densities in the initial axon segment improved action potential propagation. Modelling suggested that the single spike ﬁring to step current injection observed in whole-cell recordings is not a cellular property but a dynamic consequence of shunting resulting from electrical coupling. Without electrical coupling, firing of the population during depolarising current was unsynchronised; with coupling, the population showed synchronous recruitment and rhythmic firing. When activated instead by increasing levels of modelled sensory pathway input, the population without electrical coupling was recruited incrementally to unpatterned activity. However, when coupled, the population was recruited all-or-none at threshold into a rhythmic swimming pattern: the tadpole “decided” to swim. Modelling emphasises uncertainties about fine unmyelinated axon physiology but, when informed by biological data, makes general predictions about gap junctions: locations close to the soma; relatively small numbers; many indirect connections between neurons; cause of action potential propagation failure in fine axons; misleading alteration of intrinsic firing properties. Modelling also indicates that electrical coupling within a population can synchronize recruitment of neurons and their pacemaker firing during rhythmic activity.

## Author Summary

Some groups of nerve cells in the brain are connected to each other electrically where their processes make contact and form specialized “gap” junctions. The simplest function of electrical connections is to make activity propagate faster by avoiding the delays resulting from chemical messengers at synaptic connections. In other cases, especially in higher brain regions where more spread out nerve cells may be connected by their axons, the function of electrical coupling is less clear. To understand this type of electrical connection better we have built computer models of a group of electrically coupled nerve cells in the brain which control swimming in very young frog tadpoles. We show that the coupling can be indirect, via other members of the group, and can profoundly influence the properties of the nerve cells which would be recorded during real experiments. The main role of the coupling is to synchronise the firing of the group so they are all recruited together when the tadpole is stimulated and then fire in a rhythm suitable to drive swimming movements. The results from this simple animal raise issues which will help to understand coupling in more complex brains.

## Introduction

Electrical synapses are widespread in nervous systems from motoneurons to the neocortex and have a long history of study [1–2]. In simpler systems, they have been proposed to reduce response latency, synchronise firing, and detect input coincidence [3–8]. Electrical coupling is found within populations of similar neurons in the mammal retina [9] and brain [1] and is sometimes mediated by axo-axonic connections [10–19]. The effects of electrical coupling may be diverse [20–21], but one hypothesis is that it synchronizes activity over particular frequency ranges [22–25].

Electrical coupling is easy to demonstrate experimentally but its distribution and significance is difficult to define. Tracer dyes may reveal electrically coupled neurons and potential gap junction sites [26] but curiously, electrically coupled neurons often show no dye coupling [1]. Another difficulty is that the properties and responses of individual neurons cannot be isolated if they are electrically coupled. To investigate the effect of the gap junctions pharmacological blockers have been used but most are probably non-specific and have effects on other membrane channels [21,27–29]. Genetic knockouts are another possibility [30] but there are likely to be side-effects [31]. One approach to understanding is through modelling networks of electrically coupled neurons [32].

Our aim is to use network modelling to explore the significance of axo-axonic electrical coupling in populations of rhythmically active neurons. We have exploited the detailed evidence on frog tadpole reticulospinal descending interneurons (dINs; [29,45]) by building a model of part of the dIN population. The aim was to investigate the effects of different gap junction distributions, coupling and axon parameters, and experimental protocols. Specifically, we built a population of 30 model brainstem dIN neurons and asked: 1) can the possible locations of axonal gap junctions be inferred using passive models of their somata and axons; 2) what is the effect of axonal electrical coupling on the firing properties and axonal action potential propagation; 3) is their apparent single-spike firing a result of their electrical coupling; 4) what is the role of axonal electrical coupling in the rhythmic firing of the dIN population during swimming and their recruitment by sensory stimulation? Our results have implications for understanding the nature of axon to axon electrical coupling in fine unmyelinated axons, its role in rhythmic networks, and some of the experimental difficulties in its experimental investigation.

## Materials and Methods

This is a modelling study based on published experimental results (see first section of Results). Simulations used a custom python framework “morphforge” to model small populations of neurons [33] built on top of the NEURON simulator [34]. They were run on a desktop computer (Linux 3.2.0 on an i686 PC; NEURON (7f113b76a94b); Python 2.7)

### Evaluating gap junction distribution schemes in passive networks

The construction of the multicompartmental neuron model and networks is described in the Results. Each generated gap junction distribution scheme was evaluated by examining the distribution of coupling coefficients in a population of 30 passive neurons. The dIN axons were compartmentalised according to gap junction density, in order to maintain simulation speed (compartment lengths: 5 μm in the hillock, 5 μm for the first 400 μm of the axons and 100 μm thereafter, electrotonic length in the axon is ~250 μm). In 50 simulations, hyperpolarising current was injected into a randomly chosen source neuron (N_src_) and the steady state voltage deflections measured in all neurons. Coupling coefficients between N_src_ and all other neurons were then calculated.

### Kinetic models of active currents

Membrane potential (V) evolves according to (Eq 1). Channel kinetics were based on voltage clamp data for the currents [35–37]. The sodium (i_na_) and potassium currents (i_kf_, i_ks_) were modelled using a Hodgkin-Huxley type formulation [38]; (Eq 2) and the calcium current (i_ca_) was modelled using the Goldman-Hodgkin-Katz formulation [39–41] (Eq 3: F = 96485 C/mol, R = 8.314J/(K mol), T = 300K). The i_lk_ current is due to passive membrane leak conductance and i_ext_ is the current from a current injection protocol.

$$C\frac{dV}{dt}=i_{na}+i_{lk}+i_{kf}+i_{ks}+i_{ca}+i_{ext}$$ (1)

$$\begin{array}{l} i_{lk}=g_{lk}(e_{lk}-V)\\ i_{na}=g_{na}(e_{na}-V)m^{3}h\\ i_{kf}=g_{kf}(e_{k}-V)n_{kf}^{4}\\ i_{ks}=g_{ks}(e_{k}-V)n_{ks}^{2} \end{array}$$ (2)

$$i_{ca}=P_{ca}2\upsilon F\frac{{\left[Ca^{2+}\right]}_{i}-{\left[Ca^{2+}\right]}_{o}\;\text{exp}(-\upsilon )}{1-\text{exp}(-\upsilon )}m^{2}\;\text{where}\;\upsilon =\frac{2VF}{RT}$$ (3)

Each voltage-gated channel is gated by one or more gating variables (m_na_, h_na_, n_kf_, n_ks_ and m_ca_). Eq 4 describes the dynamics of an arbitrary gating variable X, which is governed by the steady-state and characteristic time constant functions respectively (Eqs 5 and 6). These are dependent on functions which control the opening and closing of gates gof Eq 7. The values for parameters A, B, C, D and E are given in Table 1 for each channel type. (Note that the beta-rate equation for calcium takes one of two sets of parameters depending on the membrane potential [35,36].

$$\frac{dX}{dt}=\frac{X_{\infty }(V)-X}{\tau_{X}(V)}$$ (4)

$$X_{\infty }(V)=\frac{\alpha_{X}(V)}{\alpha_{X}(V)+\beta_{X}(V)}$$ (5)

$$\tau_{X}=\frac{1}{\alpha_{X}(V)+\beta_{X}(V)}$$ (6)

$$\alpha_{X}(V),\beta_{X}(V)=\frac{A+BV}{C+\text{exp}\left(\frac{D+V}{E}\right)}$$ (7)

**Table 1:** The parameters governing the rate constants for the gating variables of the voltage gated channels in the dIN model.

Channel	Rate-function	A(ms^-1^)	B(ms^-1^ mV^-1^)	C	D (mV)	E (mV)
Ca	*α*_*m*_	4.05	0	1.0	-15.32	-13.57
	*β*_*m*_ (V<-25mV)	1.24	0.093	-1.0	10.63	1.0
	*β*_*m*_ (V>-25mV)	1.28	0	1.0	5.39	12.11
K-fast	*α*_*n*_	5.06	0.0666	5.12	-18.396	-25.42
	*β*_*n*_	0.505	0	0.0	28.7	34.6
K-slow	*α*_*n*_	0.462	8.204e-3	4.59	-4.21	-11.97
	*β*_*n*_	0.0924	-1.353e-3	1.615	2.1e5	3.33e5
Na	*α*_*m*_	8.67	0	1.0	-1.01	12.56
	*β*_*m*_	3.82	0	1.0	9.01	9.69
	*α*_*h*_	0.08	0	0.0	38.88	26.0
	*β*_*h*_	4.08	0	1.0	-5.09	-10.21

### Evaluating the effects of channel densities on firing behaviours

Initial values for channel conductances ${\hat{g}}_{X}$ (where X is the channel type) were estimated based on input resistance and current densities measured in voltage clamp-recordings from Central Pattern Generator (CPG) neurons (Table 2). The small number of channel types meant that parameter sweeps could be used to investigate the effects of channel densities on the firing properties of the neurons and the robustness of the neurons to noise. For each channel type, a set of scaling factors (M_X_) was chosen (Table 2), resulting in a parameter space of 288 possible combinations. Since the experimental recordings of dINs are from *in vivo* recordings, in which the dINs are embedded in the electrically coupled network, the simulations were performed in the same scenario. For each parameter set in the parameter space to be investigated, a network of 30 electrically coupled dINs was created, in which each dIN had conductance densities for each channel given by $g_{x}={\hat{g}}_{x}\times M_{X}\times N\left(\mu =1.0,\sigma =0.05\right)$. Normally distributed variability was introduced with a noise term (N) calculated separately for each model dIN, for each channel and for each simulation (mean = 1.0, standard-deviation = 0.05). Note that the initial leak-conductance value was calculated based on measurements of input resistance of an electrically coupled dIN *in vivo*. When multi-compartment axons were modelled with gap junctions present, we examined the effects of non-uniform channel densities on the reliability of action potential propagation.

**Table 2:** The base parameters and values of multipliers used in during the parameter sweep over channel densities.

Channel	Rev. potential	Base conductance	Values of M_X_ tested	M_X_ chosen
Calcium (ca)	-	${\hat{p}}_{ca}=0.016cm/s$ ^(a)^	0, 0.5, 1.0, 1.5	1.0
Fast potassium (kf)	-81.5mV ^(b)^	${\hat{g}}_{kf}=2.5mS/cm^{2}$ ^(b)^	0.5, 1.0, 1.5	1.0
Slow potassium (ks)	-81.5mV ^(b)^	${\hat{g}}_{ks}=2.0mS/cm^{2}$ ^(b)^	0.5, 1.0, 1.5	1.0
Sodium (na)	50mV ^(b)^	${\hat{g}}_{na}=10.0\;mS/cm^{2}$ ^(b)^	0.0, 0.5, 1.0, 1.5, 2.0, 3.0	3.0
Leak (lk)	-52mV ^(c)^	${\hat{g}}_{lk}=0.25\;mS/cm^{2}$ ^(c)^	0.5, 1.0, 1.5	1.0

^(a)^ from Dale (1995b);

^(b)^ from Winlove and Roberts (2012);

^(c)^ from Sautois *et al*. (2007), based on an input resistance of 300 MΩ. Note that this leak conductance value was calculated based on measurements of input resistance of an *in situ* dIN, which is electrically coupled.

### Modelling synaptic input to dINs following sensory stimulation

To model the synaptic input which dINs receive following head skin stimulation, we used data from whole-cell recordings made in trigeminal interneurons (tINs) which are excited by head skin sensory neurons and in turn excite dINs [42]. *In vivo* these tINs fire between 0 and 5 spikes depending on the stimulus level, so a simple model was built that generated a set of spike times for a single tIN in response to graded skin stimuli. The stimulus strength, s, is normalised so that s = 100% corresponds to a head-skin stimulus at the threshold required to initiate swimming (Experimentally, s is the normalised amplitude of current used for electrical head-skin stimulation). This model was used to drive EPSPs in the dINs to model excitation from a population of 20 tINs. In the tIN spike time model, the number of spikes fired, n, at given stimulus level, s, is generated from the probability distribution p(N = n|S = s). This simple model was based on the observations that: a) the mean threshold stimulus that leads tINs to fire a single spike is 95% (94 ± 6%), (i.e. p(N = 1|S = 95%) = 0.5); b) at 100% stimulus all tINs fire a single spike (i.e. p(N = 1|S = 100%) = 1.0); c) as stimulus strength increases above 100%, some tINs begin to fire multiply, but some always fire a single spike (10/34) (p(N = 1|S>120%) = 0.3); and d) the distributions for the number of spikes fired at high stimuli (s>120%) were estimated based on spike counts at higher stimulation levels [42]. Next, the number of spikes, n, fired by a model tIN is converted into timings, {t_1_, t_2_ … t_n_}. The time of the k_th_ spike, t_k_ is generated from a normal distribution t_k_ ~ N(μ = μ_k_, σ = σ_k_), where μ_k_ and σ_k_ are the means and standard deviations of the k'th spike. The values of μ_k_ and σ_k_ were calculated from experimental data taken at all levels of stimulation [43].

## Results

### *Xenopus* tadpole reticulospinal neurons

**Fig 1:**
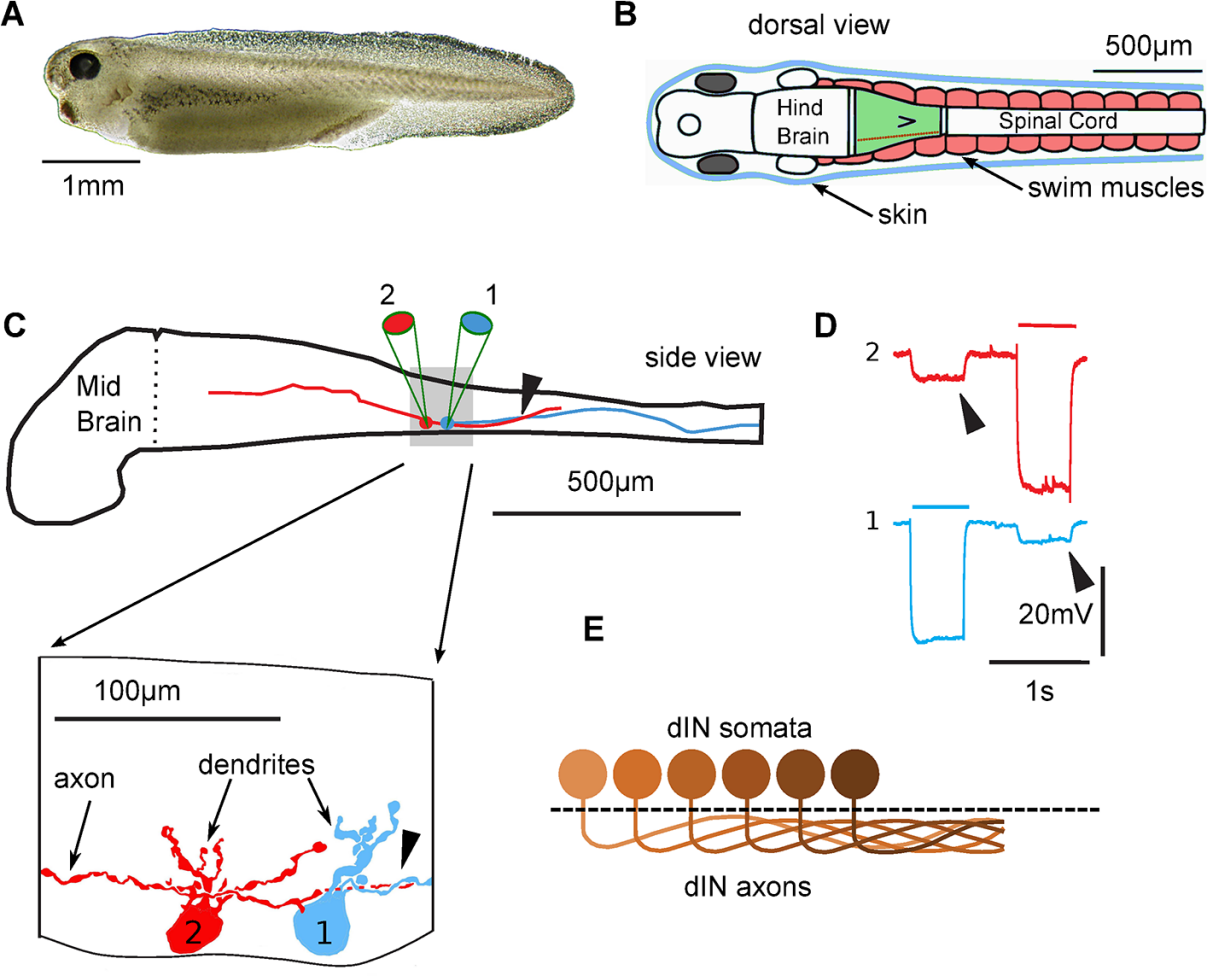
The hatchling tadpole CNS and a population of electrically coupled neurons (dINs). (A) Side view of a hatchling *Xenopus laevis* tadpole 48 hours after fertilisation. (B) Top view diagram of the tadpole showing skin, swimming muscles, and CNS with hindbrain and spinal cord. The CNS region able to generate swimming rhythm when isolated (green) contains a population of ~30 dIN neurons (brown) on each side. (C) Side view of the hindbrain and spinal cord showing diagrammatic recording electrodes (1 and 2) and tracings of two filled dINs (from [29]) with somata and short dendrites. dIN-1 has only a descending axon (arrowhead). dIN-2 also has an ascending axon (arrow). The intertwining of the axons can be seen (arrowheads). (D) Electrical coupling was shown using simultaneous voltage recordings from two dINs. Hyperpolarising current injection into either dIN (bars) caused a small voltage deflection in the other (arrowheads; from [29]). (E) Diagram of part of the dIN column with descending axons leaving the somata and intermingling where axo-axonic gap junctions could lie.

To investigate the effects and significance of electrical coupling in a relatively simpler network we have studied small columns of neurons in the brain of hatchling frog tadpoles. Two days after fertilization, tadpoles of *Xenopus* laevis are approximately 5 mm in length (Fig 1A) and respond to a brief touch stimulus by swimming for several seconds [44]. The locomotor circuits generating swimming rhythms consist of ~2000 neurons divided into <10 classes. These neurons form longitudinal columns on each side, starting in the hindbrain and descending into the spinal cord. Lesion studies have shown that a small region of the nervous system, 0.3 to 0.4 mm long in the hindbrain and rostral spinal cord (Fig 1B), is sufficient to produce sustained swimming-like rhythms in response to stimulation [45]. One class of reticulospinal neuron, descending interneurons (dINs), is present in this region with a population of about 30 on each side. These neurons play a central role in the generation of swimming rhythms and directly excite motoneurons [46]. Whole-cell recordings showed that the dINs are the first neurons to fire on each side on each cycle of swimming, and concluded that they are the neurons which drive swimming [47]. Recordings from pairs of these dINs up to 200 μm apart in the longitudinal column on one side have shown that they are electrically coupled (Fig 1C and 1D; [29]). In some cases dIN dendrites extend far enough longitudinally to allow short range coupling (Fig 1C neuron 2) but more usually their dendrites have very limited longitudinal extents (< 30 μm; Fig 1C neuron 1). This means that coupling between dINs from 50 to 200 μm apart is likely to be via contacts between their very thin (<0.5μm), unmyelinated, descending longitudinal axons (Fig 1C). After emerging from the soma these axons lie close to each other for a while before they diverge (Fig 1E) and could therefore make gap junctional connections. When gap junction blockers were applied, swimming episodes became shorter and dIN firing became unreliable, suggesting that the electrical coupling is important for reliable rhythm generation. Curiously, whole-cell recordings from dINs show they only fire once in response to step-current injection. This property may be important for network mechanisms of rhythm generation based on Post Inhibitory Rebound (PIR; [48]). This single firing response seems at odds with the repetitive, 20 Hz pacemaker firing seen when the whole dIN population is excited by the experimental application of excitatory synaptic agonists like glutamate [49]. Glutamate activates NMDA-receptors (NMDARs) which play a fundamental role when dINs drive swimming [45].

### A passive population model of electrically coupled neurons

**Fig 2:**
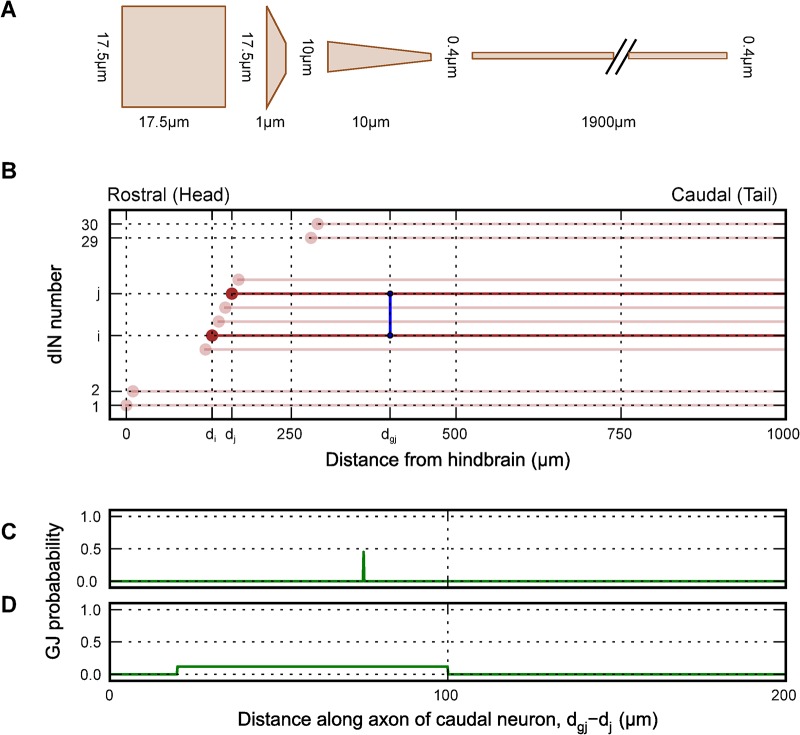
A passive population model of descending interneurons in the hindbrain and rostral spinal cord and their gap junctions. (A) dIN morphology was approximated as four sections each modelled as a cylinder or tapered cylinder, one for the soma, two for the hillock and one for the axon (not to scale). (B) The somata of the dINs form a rostral-caudal column (light: circles = somata) each with a descending axon (lines). In this example, there is a single gap junction (dark vertical line) between a pair of dINs {i, j}. (C, D) Two layout schemes for generating gap junctions between two axons were tried, to evaluate their effects on coupling coefficients: (C) A gap junction could occur with a certain probability at a fixed point along the axon of the more caudal dIN; (D) A gap junction could occur with a certain probability at any location within a defined region of the axon of the more caudal dIN.

The electrical coupling between reticulospinal dINs in the tadpole hindbrain is likely to be axo-axonic because only their axons are long enough to allow contacts at the longer distances between coupled somata [29]. It is possible that coupling could be axo-dendritic or axo-somatic or via ascending dIN axons when these are present but to simplify our analysis we have not considered these cases. The strength of coupling between two dINs, measured at their somata, will therefore be determined by the resistance of the gap junctions, their positions along the axons and the axon properties. The effects of neuronal morphology were taken into account by building multicompartmental models of dINs which included an axon. dINs have a soma with short dendrites, which together can be considered as a single isopotential compartment [50], and a single thin axon (<0.5μm) which descends towards the tail for 280 to 2050 μm [51]. In some cases hindbrain dINs also have an ascending axon (Fig 1C). The surface area of dIN soma plus dendrites was estimated as ~1000 μm^2^ from tracings of motoneurons with very similar size and morphology. This is a round number corresponding to a sphere of diameter ~17.5 μm and a small taper into the axon. (It lies within the range of summed soma and dendrite surface areas measured for filled motoneurons: 754 to 1594 μm^2^; n = 5) A multicompartmental model was built with four sections to represent dIN morphology: one for the soma-dendrites, two for the hillock and one for the axon (Fig 2A). The shortness of the dendrites means that they are equipotential with the soma and can be modelled as a single compartment (see: [50]). The axon compartment was further compartmentalised during simulations (see below). These smaller compartments are electrically connected together via resistances, which are calculated based on the intracellular resistivity, Ri, of the neuron, and the surface area that connects the two compartments (mathematics described further in [34]). Initially, the model dINs were given a uniform leak conductance (g_lk_) over their surface, to match the dIN input resistance measured experimentally, an intracellular resistivity R_i_ = 80 Ω cm and a specific capacitance C = 1.0 μF/cm^2^ [41]. The somata of the dINs driving swimming form a longitudinal column. The axons of dINs grow into the marginal zone of axons surrounding the spinal cord then grow towards the tail (Fig 1C and 1E; and Fig 2B), so they could contact dendrites or axons of other dINs. Based on experimental measurements of cell distributions and reconstructions from fills with neurobiotin [45,51], we created a column of 30 model dINs in the hindbrain and rostral spinal cord, with somata spaced at 10 μm intervals.

**Fig 3:**
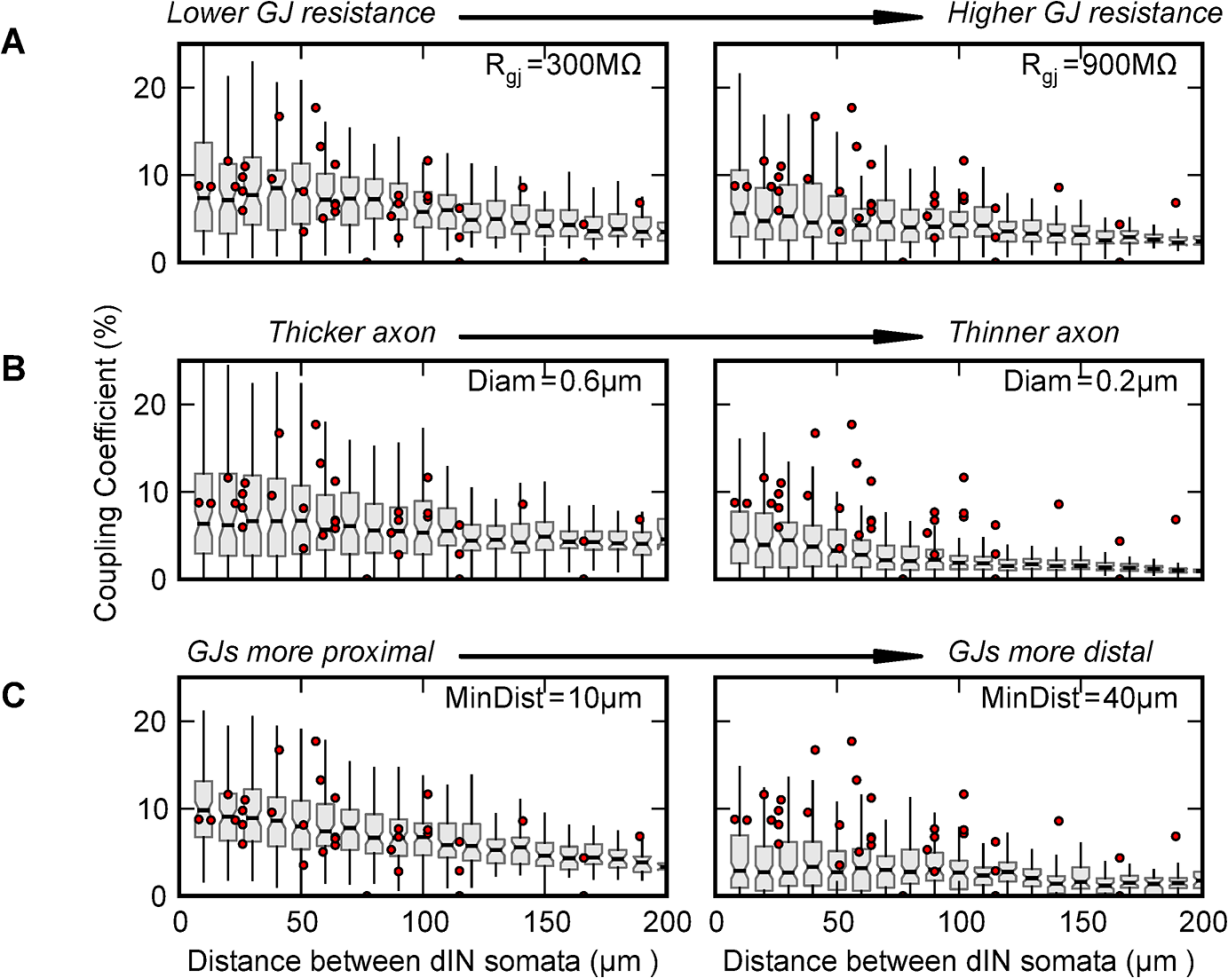
The effects of different parameters on the coupling coefficients measured between neurons in a passive population model. In (A-C) a single parameter is varied (two values are shown) and the coupling between model neurons at different distances was measured for 100 generated networks for comparison with the coupling between pairs of dINs measured experimentally (shown by dots in each graph; [29]). The notched grey bars show the median and 25% to 75% quartiles of the model coupling coefficient at each distance. The lines represent the 5% to 95% percentiles. Starting parameters (see text): R_i_ = 80 Ωcm, R_GJ_ = 600 MΩ, min-dist = 20 μm, max-dist = 70 μm, g_lk_ = 0.25 mS cm^-2^ and axon diam = 0.4 μm. In each case the visual match to experiments is better in the left column than in the right column. (A) R_GJ_ reduced from 600 to 300 MΩ or increased to 900 MΩ. (B) Axon diameter increased from 0.4 to 0.6 μm or reduced to 0.2 μm. (C) Minimum distance from the caudal soma was reduced from 30 to 10 μm or increased to 40 μm. The parameters min-dist and max-dist correspond to the left and right-hand edges of the GJ distribution step in Fig 2D respectively. When gap junctions were distributed closer to the somata of the neurons, the coupling was stronger (in part because there are more gap-junctions in the network in total).

**Fig 4:**
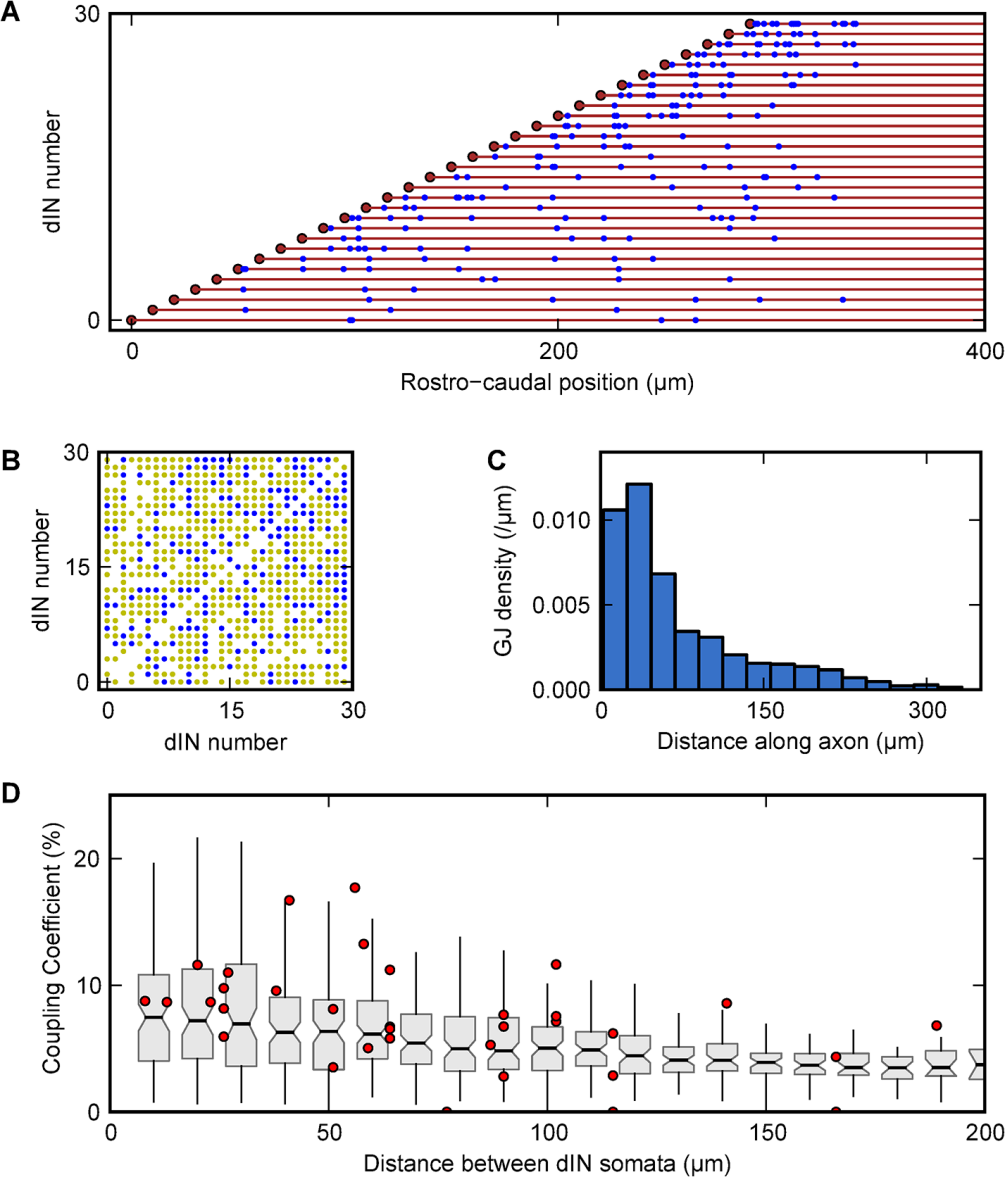
Gap junction distribution and coupling coefficients between dINs. (A) The chosen layout of gap junctions in the column of 30 dINs (somata red circles spaced 10 μm apart) with descending axons (red lines). Blue dots represent one side of a gap junction (hemichannel), which connects to another gap junction at the same position along the column (x axis). (B) Direct and indirect dIN to dIN coupling: directly coupled neurons (blue dots); indirectly coupled via the axon of another neuron (yellow dots); coupled via more than 3 axons (empty square). (C) Histogram of gap junction distributions along the axons of dINs. In order to get good coupling between dINs, we found that gap junctions needed to form close to the somata. (D) Comparison of the coupling coefficients between pairs of dINs measured experimentally (red circles; [29]) and in the model (as in Fig 3).

In the tadpole, current injections during paired whole-cell patch-clamp recordings have shown electrical coupling between dINs with somata up to nearly 200 μm apart [29]. The coupling coefficient is defined as the change in potential of a coupled neuron expressed as a percentage of the change in potential of a neuron into which hyperpolarising current is injected. The coupling coefficient was found to decrease with distance between the two neurons, from ~10–15% (at 0–50 μm) to ~5% (at 150–200 μm; Figs 3 and 4D). In paired recordings from dINs in normal saline, we injected depolarising current to evoke an action potential. In no case did such action potentials lead to firing in unstimulated dINs or to motoneuron excitation [45]. The location of the gap junctions responsible for coupling was not established experimentally but detailed dIN anatomy is available from single neurons fills [51]. If the dINs are very close to each other (say <50 μm), gap junctions could be dendro-dendritic. If they are further apart the main possibility is axo-dendritic or axo-axonic gap junctions. Since the dendrites are electrotonically compact, both dendro-dendritic and axo-dendritic coupling can be seen as a special case in which the distance along the axon from the soma-dendrite compartment was 0 μm. To simplify our study and avoid further assumptions, we only considered axo-axonic coupling between dINs with descending axons. An advantage of using the dINs in the *Xenopus* tadpole to study axo-axonic coupling is that the layout is constrained to a single longitudinal dimension; the location of a gap junction coupling two dINs can be expressed simply as the distance of the gap junction from the more caudal dIN in the column (d_gj_ in Fig 2B).

### Inferring the locations of axonal gap junctions using passive models

We investigated how the numbers, strengths and locations of gap junctions with simple values of the gap junction resistance (R_GJ_) would affect the electrical coupling measured between the dINs within the model column (Fig 2B). Anatomical studies indicate that dIN axons intermingle in the marginal zone for a few hundred microns after emerging from the soma. Where axons overlapped, model gap junctions therefore had the same probability of forming (per unit length) between any two axons. The layout of the network leads to more gap junctions forming caudally, because the axons descend. For a bundle of parallel, cylindrical axons of equal diameter, one axon can be in contact with a maximum of six others at any point. Therefore at each point of each axon, we limited the possible connectivity to a maximum of six neighbouring axons but as new axons add to the column different axons will be able to make contacts with each other. The simplest layout scheme of gap junctions between pairs of overlapping axons which is consistent with the anatomy is for the axon of a more rostral dIN to form junctions with the axon of a more caudal neuron at a fixed position from the caudal soma with a certain probability (Fig 2C). However, this type of layout led to local clustering of gap junctions with consequent large shunting which would cause axonal action potential propagation failure (see below). We therefore used the next most simple layout with a fixed gap junction probability density over a defined region of the axon of the more caudal dIN (Fig 2D).

Gap junction distribution schemes were evaluated by examining the distribution of coupling coefficients in a population of 30 passive neurons. The dIN axons were compartmentalised according to gap junction density in order to maintain simulation speed (compartment lengths: 5 μm in the hillock, 5 μm for the first 400 μm of the axons and 100 μm thereafter; electrotonic length in the axon is ~250 μm). In 50 simulations, hyperpolarizing current was injected into a randomly chosen source neuron (N_src_) and the steady state voltage deflections measured in all neurons. Coupling coefficients between N_src_ and all other neurons were calculated and plotted against the distance between somata (Figs 3 and 4D). The small number of parameters in the system meant that each layout strategy could be tested using a parameter sweep through different values of g_lk_ (0.1 to 0.5 mS/cm^2^; [48]), R_i_ (40 to 150 Ωcm; [41]), axon diameter (0.1 to 0.6 μm; [52]), min-dist (0 to 100 μm), max-dist (0 to 100 μm), and R_GJ_, (50 to 2000 MΩ; [23]). Model networks were evaluated by visually comparing the distribution of their coupling coefficients as a function of distance with those found experimentally [29].

Using the fixed probability density layout for gap junctions (Fig 2D), we explored the parameter space influencing the distribution of coupling coefficients to find areas where there is a good fit to the experimental results (Fig 3). Some areas produced distributions that were clearly inappropriate. For example, raising the gap junctional resistance weakened coupling at all distances (Fig 3A). When the diameter of the axons was 0.3 μm or less, the fit of the model to experiments was poor and large areas of parameter space produced low coupling coefficients between distant neurons compared to experimental observations (Fig 3B). To get good coupling over distance, gap junctions needed to be less than 50 μm from the soma of the more caudal neuron (Fig 3C). Interestingly, when the coupling was too close (a substantial proportion of the gap junctions within 10 μm of the caudal soma), neurons closer together showed stronger coupling but the strengths of coupling over long distances were also reduced. Some care must be taken interpreting these results. In general, changing parameters in one way, (for example moving gap junctions proximally, or decreasing their resistance) leads to stronger coupling in the network, while others, (such as reducing the numbers of gap junctions) weakens coupling—often changing one parameter can be offset by changing another.

Further exploration of gap junction distribution in coupled networks was based on results from our parameter sweeps. These showed that a gap junction layout scheme with a fixed probability density over a defined region, (Fig 2D) gave coupling coefficients similar to those observed experimentally, and was biologically plausible. Gap junctions were therefore generated between overlapping axons with a fixed probability density of 0.015 μm^−1^ over a region of the caudal axon starting at the minimal distance (min-dist) and finishing at the maximal distance (max-dist; Fig 2D). To implement this the first 50 μm region of each axon was divided into 1 μm bins and for each bin, six other dINs with axons at the same position along the rostrocaudal axis were picked at random and given a fixed 0.015 probability of forming a gap junction. A gap junction resistance of 600 MΩ gave coupling coefficients similar to those measured experimentally. This method generated 80–120 gap junctions in the network of 30 dINs, which means each axon has about 5 to 8 gap-junction connections (Fig 4). The small number of connections on each axon resulted in only 25% of neuron pairs being directly coupled (Fig 4B; blue dots). Most pairs were indirectly coupled via the axon of one other neuron (Fig 4B; yellow dots). We found that when a dIN was electrically coupled in the network, its input resistance, calculated by injecting hyperpolarising step current, dropped by ~50%. To restore the model dIN input resistance to match values measured physiologically in dINs coupled to their neighbours [29], the leak conductance (g_lk_) of the neuron was decreased by 50% to 0.125 mS/cm^2^.

### Adding active channels to the model neurons and their axons

To build active models of the network of dINs we needed to match their unusual properties. The dINs play a critical role in driving the firing of other neuron types on the same side of the CNS during swimming as they are the first neurons to fire on each side on each cycle [47]. Whole-cell recordings have shown that dINs have an unusual response to a step current injection and only fire a single action potential, even at levels of injected current up to twice threshold [45]. Furthermore, it has been proposed that dIN action potentials during swimming are partly the result of PIR following inhibition from the opposite side of the spinal cord [45,53]. Rebound firing is not seen when dINs are hyperpolarized from rest but only during swimming when they are depolarised [44]. In line with this, while dINs are depolarised by injected current, short hyperpolarizations or Inhibitory Postsynaptic Potentials (IPSPs) can lead to rebound firing [47]. The densities and kinetics of the voltage-gated channels play an important role in determining the firing properties of neurons [54]. In *Xenopus* tadpole spinal neurons, voltage clamp experiments on dissociated neurons [36] and neurons *in situ* [55] have suggested the presence of about 8 types of voltage-gated ion channels. In dINs, the majority of the voltage-gated currents are thought to be carried through sodium channels (Na), fast and slow potassium channels (Kf, Ks) and high-voltage-activated calcium channels (Ca). The kinetics of these channels are described in the Methods section.

**Fig 5:**
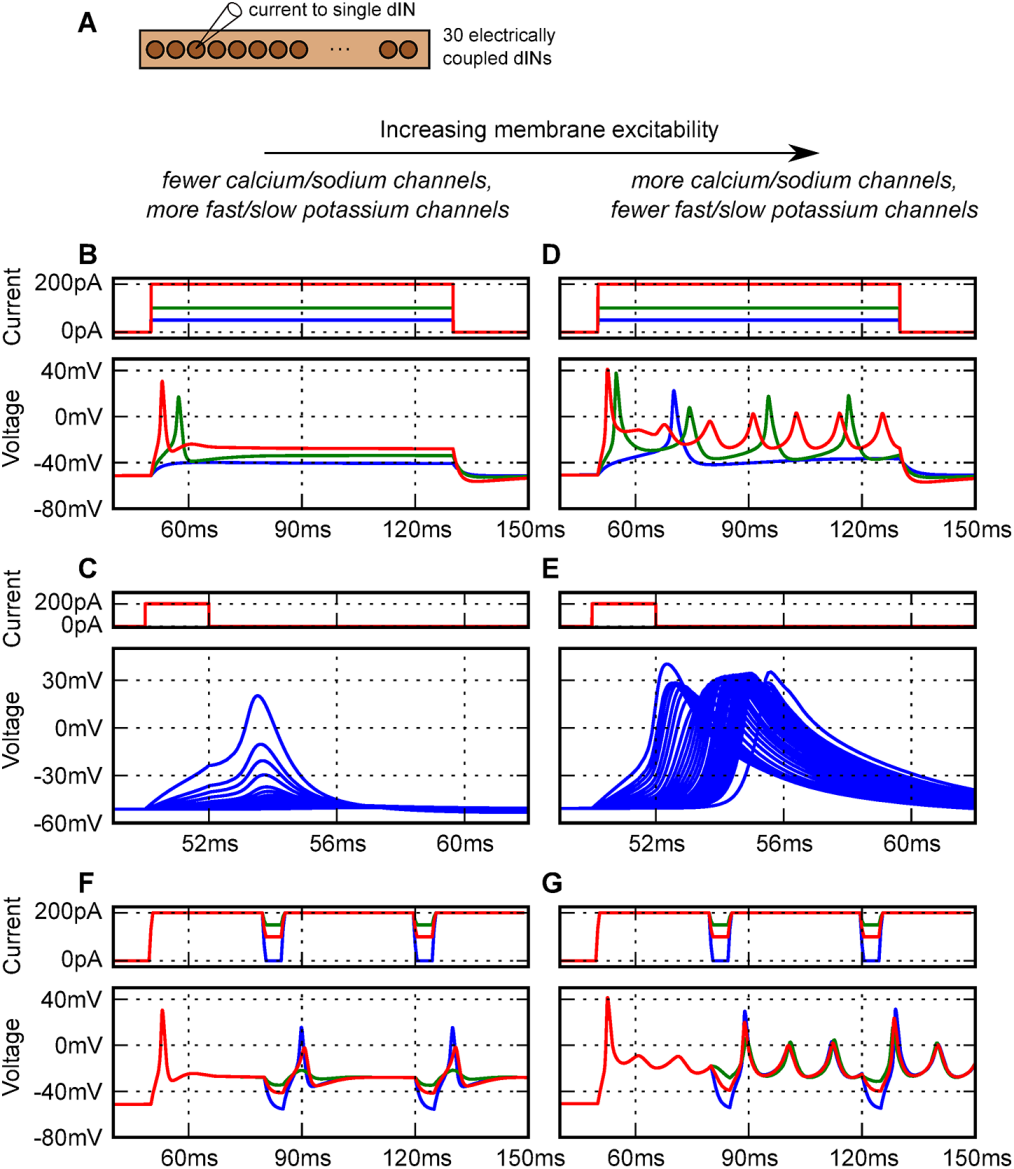
Effects of uniform changes in densities of channels on model dIN membrane excitability, firing and action potential propagation. (A) Current was injected into a single dIN in a population model of 30 dINs electrically coupled via axo-axonic gap junctions. (B, C) When the membrane excitability is low, model dINs reliably fire a single action potential at the onset of current injection (B) but would not reliably conduct action potentials along their axons (C). (D, E) With increased excitability, the model neurons fire repetitively to higher levels of current injection and (E) action potential propagation could be reliable. (C, E) Action potential propagation in the model was tested by recording the voltage in the soma and at 20 points along the axon (20 μm spacing) of a random neuron in the network. A short current pulse injected into the soma is used to initiate an action potential (first trace). (F, G)**.** Rebound firing was investigated using short hyperpolarising step current pulses during a longer depolarising step current injection. (The values for these traces were (B, C, F): M_ca_ = 0.5, M_na_ = 1.0, M_kf_ = 1.0, M_ks_ = 1.5, and M_lk_ = 0.5 and (D, E, G): M_ca_ = 1.0, M_na_ = 1.5, M_kf_ = 0.5, M_ks_ = 0.5, and M_lk_ = 0.5).

The responses of model dINs and their axons to current injection were evaluated in the electrically coupled population using the layout scheme and parameters found above. For each channel type (X) we used an estimate for its conductance density (${\hat{g}}_{x}$) and created a set of possible open-channel conductances: $g_{x}={\hat{g}}_{x}\times M_{X}$, where values of *M*_*X*_ were a chosen set of multipliers for each channel type (e.g. *M*_*lk*_ = (0.5, 1.0, 1.5); further details are given in the Methods section). We used a parameter sweep (details in the Methods section) over these channel densities and evaluated three responses at every point in the parameter space: a) whether the model neuron reliably fired only a single action potential in response to step current injections of 50, 100, 200 and 300 pA; b) whether an action potential initiated in the soma would propagate along the axon; and c) whether the model neuron would ‘fire-on-rebound’ to short, hyperpolarizing current injections given during step depolarizing current injections. These are all typical and identifying features of dINs [45]. Since variability is introduced into model parameters (see below), 10 neurons were evaluated at each point. In the first parameter sweep, channels were applied uniformly to the neurons and axons. In general increasing Na and Ca channel density and reducing K channels increased the excitability of the neurons (Fig 5). With fewer Na and Ca channels but more K_f_ and K_s_ channels dINs robustly fired only a single action potential (Fig 5B) but there was a problem. Action potential propagation along the axon (Fig 5C) only occurred in < 10% of cases. With more Na and Ca channels but fewer K_f_ and K_s_ channels, dINs fired once at threshold (rheobase) but fired repetitively as current was increased (Fig 5D). Action potential propagation occurred (Fig 5E) but only in 20% to 40% of tests. At high densities of Na and Ca channels, rhythmic firing could continue after the current injection or in some cases even without any stimulation. For most sets of parameters, some form of rebound firing occurred when sufficient hyperpolarising current was injected while the dIN was depolarised (Fig 5F and 5G).

Can changes in gap junction resistance or the distribution of voltage-gated channels in the axon make action potential propagation more reliable? When gap junction resistance was increased from 600 MΩ to 2000 MΩ, action potentials were always able to propagate, suggesting that the failure to propagate was due to the shunting of current through the gap junctions. In other fine, unmyelinated axons, densities of sodium channels up to 50 times those found elsewhere have been observed in the initial segment of the axon close to the soma [56–59]. To increase the proportion of action potentials propagating over regions with 600 MΩ gap junctions, without increasing the overall excitability of the model dINs, we carried out a parameter sweep in which channels were not distributed evenly over the neuron. Initially, we changed the density of Na and K channels only in the axon. Although increasing Na channel density by 200% and reducing K by 50% improved action potential propagation to ~40%, it also produced networks that were unstable, and fired repetitively without any input. The next step was to adjust channel densities only in the initial region of the axon between 20 and 70 μm from the soma. Increasing Na channel density in this region by 5 to 10 times made action potential propagation more reliable without the network becoming unstable. Increasing the Na channel density by a factor of 5 improved action potential propagation rates to over 50%. *In vivo*, dINs are usually active together so the current leak to other dINs will be less than when a single dIN is active alone and propagation should be reliable (see below). However, recordings from pairs of neurons *in vivo*, showed that stimulating a single dIN to fire reliably produced an excitatory post-synaptic potential in the other neuron which implies that dIN axonal action potential propagation was reliable [45]. There is therefore a mismatch with the model.

**Fig 6:**
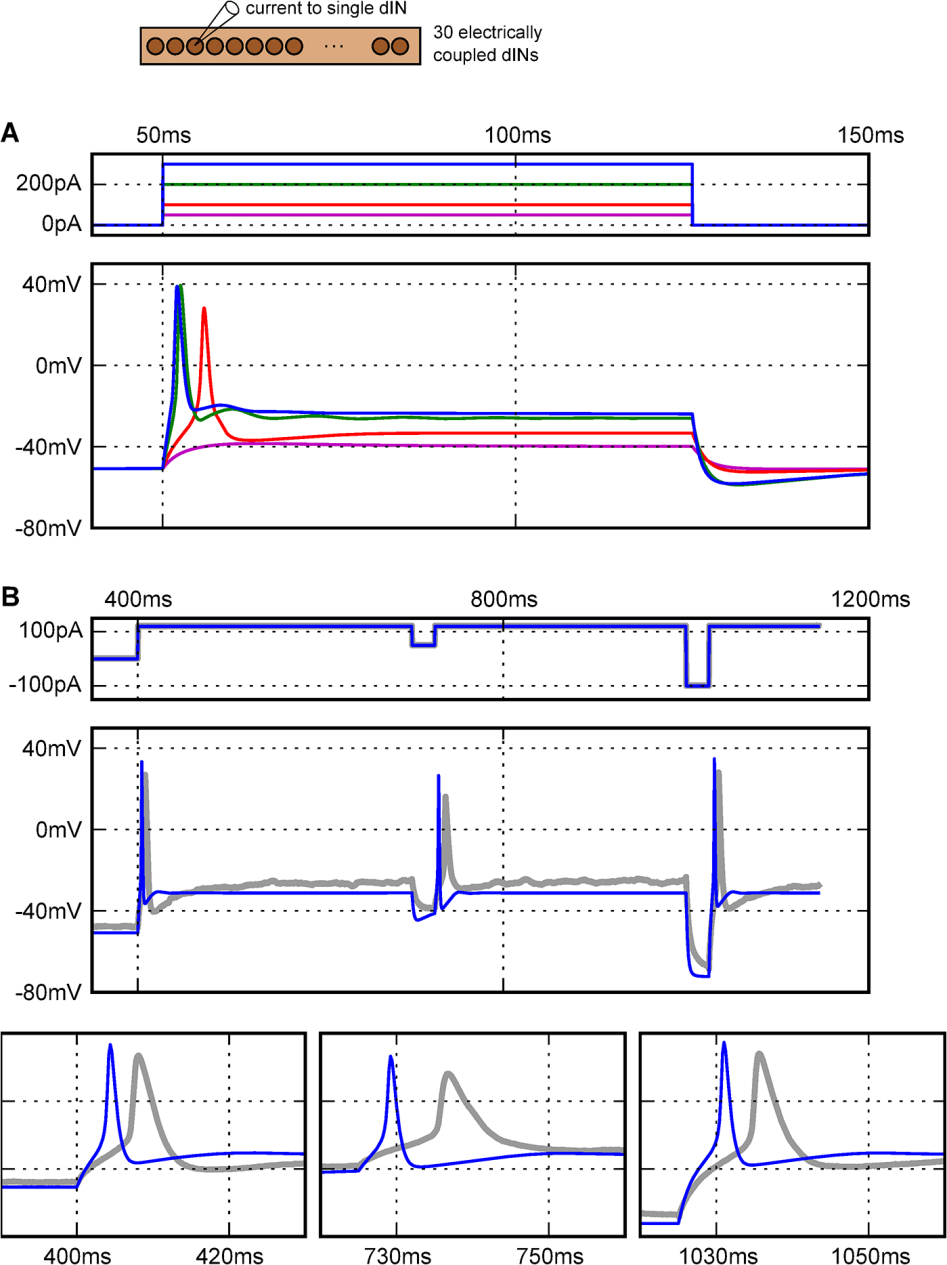
Responses of final active dIN model to current injections and comparison to physiological recordings. (A) In response to increasing levels of step current injection (top), the model neuron soma has a firing threshold of ~80 pA (red), and fires a single action potential, even in response to currents of 3 times the threshold (blue). (B) Rebound spikes to negative current steps (expanded in bottom traces) during depolarisation in model neurons (thin blue line) and whole-cell recordings (thick grey line; from Dr Wen-Chang Li, unpublished).

The parameters for the final dIN model were selected so its properties were as close as possible to those observed in whole-cell recordings of real dINs (Fig 6; cf [45,47,48]): input resistance 300 MΩ; firing threshold 80 pA; action potentials with broadly similar rise times and durations; single firing in response to large step current injections (> 300 pA; Fig 6A); and firing on rebound in response to short hyperpolarising current injections during a long depolarising step current injection (Fig 6B). The rise time and duration of the model action potential to current injection were shorter than those seen in recordings. The rise-time could be increased by the addition of an A-type potassium channel [54] but since its introduction had little effect on network dynamics, it was not included. Action potentials propagated more slowly over regions of gap junctions (~0.16 m/s). When gap junctions were removed the conduction velocity was ~0.32 m/s. These values are similar to conduction velocity estimates of 0.12± 0.27 m/s for the central axons of excitatory spinal neurons in *Xenopus* which may be dINs [60].

### Does electrical coupling influence the properties of neurons determined during recordings?

**Fig 7:**
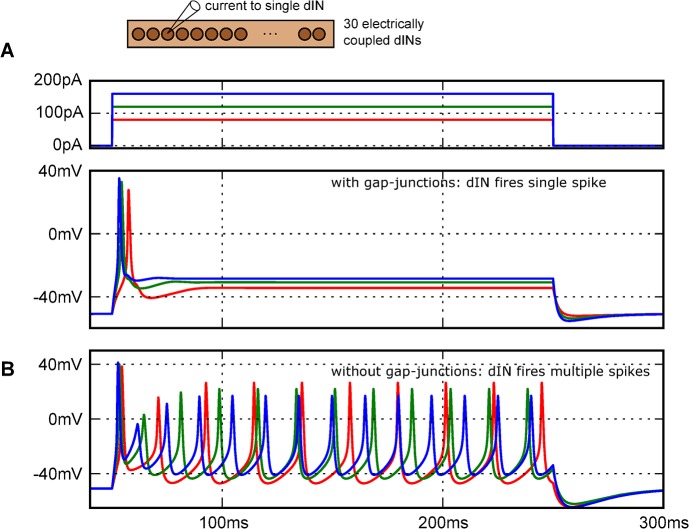
Effects of electrical coupling on firing properties of single stimulated dINs. (A) Diagram of network of 30 electrically coupled dINs where injection of 200 ms step current at increasing levels (red, green, blue) into a single dIN reliably produces only a single spike. (B) When gap junctions were removed, the injected dIN fires repetitively.

We would expect that properties determined using whole-cell recording electrodes are influenced by neurons being electrically coupled to other neurons. We therefore investigated whether the limited excitability and single spike firing of tadpole dINs was a result of the current sink effects of electrical coupling. We compared the response of a model dIN to step current injections in an electrically coupled network, and in isolation (Fig 7). When electrically coupled within the network, dINs fired only once to all levels of injected current (Fig 7A). Removing the coupling between dINs had two effects. The input resistance of individual dINs recorded at the soma increased (from ~300MΩ to ~600MΩ) and the same step current injections now caused them to fire repetitively (Fig 7B). The simplest interpretation of this effect is that dIN excitability is normally depressed by current flow through gap junctions from the stimulated neuron to its neighbours which sit at a more negative membrane potential, closer to rest. These results have significant implications for the interpretation of recordings from electrically coupled neurons and for conclusions based on the use of pharmacological agents to try to block electrical coupling (see #sec_discussion).

### What is the effect of axonal electrical coupling on network firing responses?

**Fig 8:**
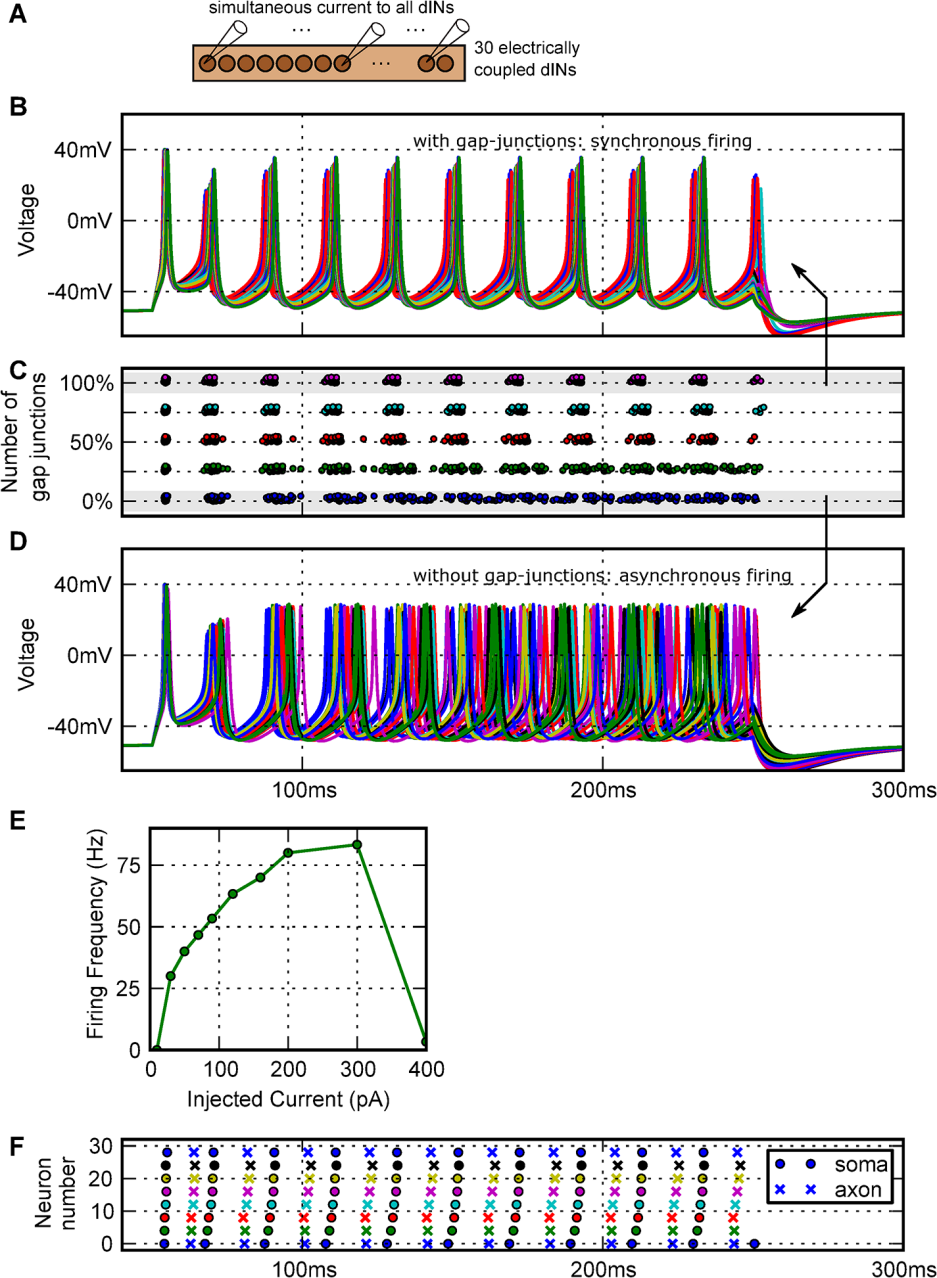
The effects of electrical coupling on firing of a population of 30 electrically coupled dINs. (A) Current is injected into all the electrically coupled dINs. (B) Even at threshold, the whole population fires repetitively, and in synchrony. (C) Raster plots of spike times show that as the number of gap junctions in the network was reduced, neurons still fired repetitively but synchronisation decreased. (D) Firing becomes desynchronised with 0% gap junction coupling. (E) Current-frequency curve for a dIN in the electrically coupled network. (100% gap junction coupling, equal current injected into all dINs). (F) When the dIN network with 100% coupling is synchronously active, a raster plot of every fourth dIN shows spikes recorded in the soma (circles) propagate reliably for 1500 μm along the axon (crosses; network setup identical to B).

Our modelling led to the hypothesis that the single spiking response seen physiologically to step current injection into a single recorded dIN is caused by shunting of current into nearby electrically coupled dINs whose membrane voltages remain close to rest. To test this hypothesis, we injected a 200 ms step current pulse simultaneously into all 30 dINs in the electrically coupled network. At threshold, the dINs did not fire singly but the whole dIN population fired nearly synchronously and repetitively at ~30 Hz during the current injection (Fig 8A). As the current was increased, the firing frequency increased to ~80 Hz, before repetitive firing failed at higher current levels (Fig 8E). This peak frequency is considerably higher than the mean firing frequencies seen when dINs *in vivo* are perfused with glutamate or NMDA (~17 Hz; [49]). The effect that the gap junctions played in synchronizing network firing was investigated by reducing the number of gap junctions in the network to 0, 25, 50 or 75%. As the level of coupling was reduced, firing became less synchronised revealing the variability in the dIN population (Fig 8C and 8D). When the whole model network fired synchronously, action potentials in each dIN propagated reliably for 1500 μm along their thin axons (Fig 8F).

### What is the effect of electrical coupling on neuron recruitment by synaptic excitation?

**Fig 9:**
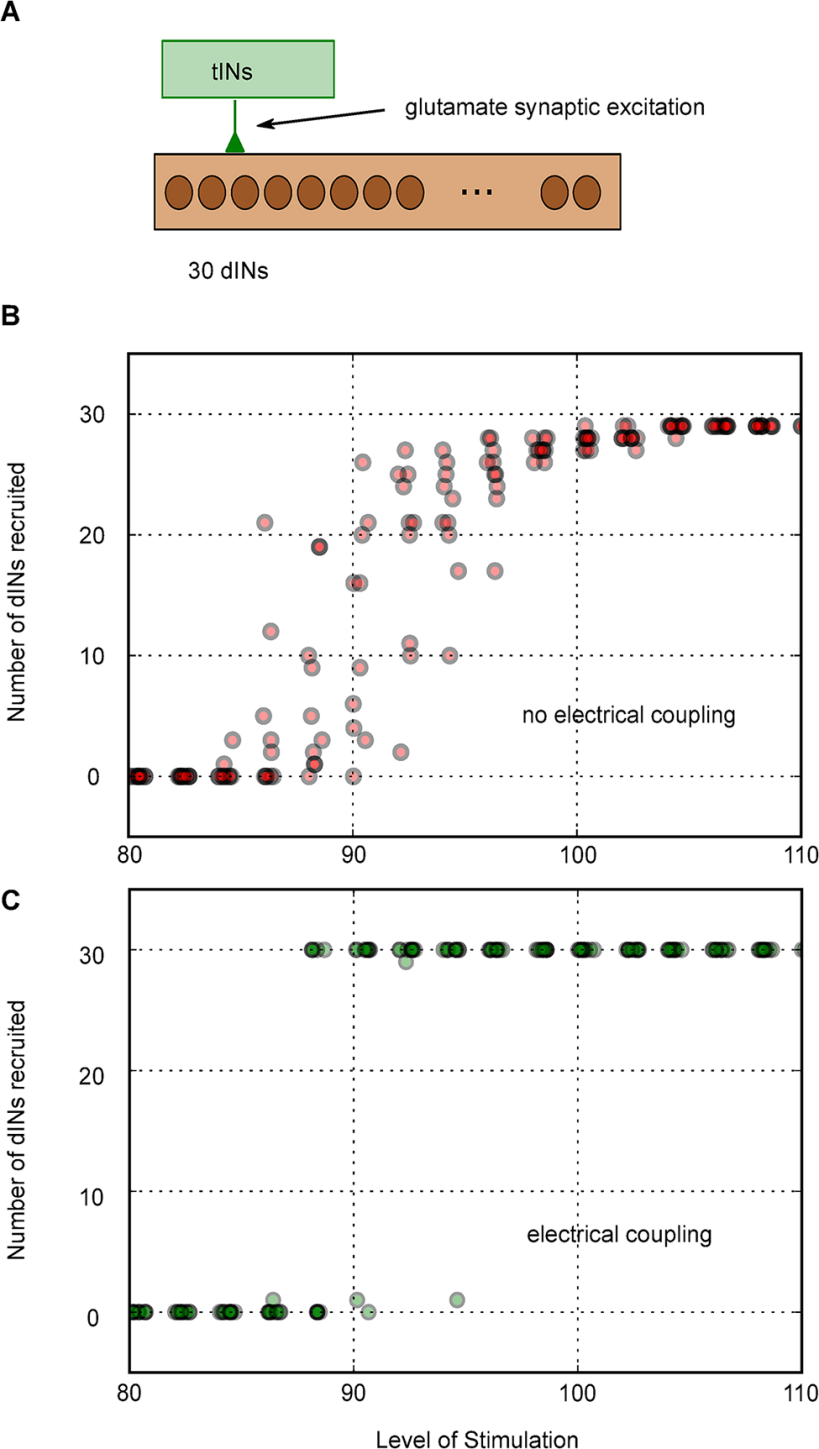
The effects of electrical coupling on recruitment of dINs by synaptic excitation following head skin stimulation. (**A**) 20 sensory pathway tIN interneurons produce glutamate EPSPs in the dINs in a pattern based on recorded responses to head skin touch (see Methods). (**B**) Without dIN electrical coupling, more individual dINs are recruited as the stimulus is increased. (**C**) With electrical coupling, there is a step recruitment of the whole dIN population. A ‘Level of Stimulation’ of 100 is defined as one that will reliably initiate swimming measured experimentally (see Methods).

The evidence from whole-cell recordings suggests that tadpole reticulospinal dINs have to be recruited as a population before swimming can start [42,47]. Does their electrical coupling affect the way in which this dIN recruitment happens? To investigate this question we modelled the pathway for excitation of dINs following head skin stimulation [42]. The trigeminal sensory neurons innervating the head skin on one side directly excite a small population of approximately 20 trigeminal interneurons (tINs) which, in turn, directly excite dINs at glutamatergic synapses. As the stimulus to the skin increases, more tINs are recruited, they fire more action potentials and their EPSPs sum in the dIN population. We have defined this pattern of summation of tIN EPSPs in dINs as a function of skin stimulus strength (see Methods). This means that we can now use it to study how the 30 dINs in our simple model population are recruited as stimulus strength to the head skin increases (Fig 9). Without electrical coupling a few dINs are recruited at low stimulus strengths and the number gradually increases with the stimulus but only slowly approaches 100% firing at higher stimulus strengths (Fig 9B). In contrast, when the dINs are electrically coupled, a higher stimulus strength is required to recruit the first dIN but, above this level, the whole population is recruited together (Fig 9C). Electrical coupling therefore makes the dIN population act like a switch which is either off (at rest) or fully on (during swimming).

## Discussion

Electrical coupling is widespread and proposed to play many roles in nervous systems, from ionic transport between cells [61], to increasing response speed [3], synchronising action potentials and contributing to computation [62,63]. Our modelling of axo-axonic coupling within a linear column of tadpole brainstem reticulospinal neurons raises some important questions. These concern: 1) the axonal distribution of gap junctions; 2) the effect of axonal electrical coupling on the firing properties and axonal action potential propagation in neurons with fine unmyelinated axons; 3) the experimental techniques used to investigate electrical coupling and its significance; and 4) the role of coupling in the operation of networks controlling rhythmic motor output, like locomotion. We will now consider these questions in turn after some brief caveats. Modelling can only be accurate and trustworthy when it is based on good evidence. We hope that we have made it clear where this is the case and where it is not. Unfortunately, there is uncertainty about the locations of gap junctions which could also be made onto dendrites or somata. Anatomical methods are required to resolve these uncertainties. There is also uncertainty about the distribution and densities of membrane channels in both the soma and fine unmyelinated axons of the reticulospinal dINs which we have modelled. Better models must depend on obtaining better evidence. Despite this, we hope that our study provides some valuable insights into the operation of a well characterised population of brain neurons [46].

### Effects of gap junction distribution on axo-axonic electrical coupling

To date, electrical coupling has been described between diverse neurons types in the mammal brain but the evidence is not extensive and sometimes contentious (see: [1,2,21]). This coupling is often fairly local, within distances of ~200 μm, but determining how many gap junctions are made by individual neurons and how their geometry is organised has proven difficult especially where networks are 3-dimensional. We have shown here how modelling can help. Fortunately, the tadpole dIN neurons which we have studied form a relatively simple longitudinal column in the hindbrain. Since their reliable electrical coupling acts over distances up to 200 μm and they usually have very short dendrites, it is most probably via gap junctions between their descending longitudinal axons [29]. Only detailed anatomical study with immune-labelling will resolve the true location of the junctions. Our modelling suggests firstly, that gap junctions need to be within 30 μm of the soma to get a realistic distribution of coupling coefficients as a function of distance between neurons that matches measured values, and secondly, that about 80% of coupling is indirect (Fig 4B). Paradoxically, adding gap junctions did not necessarily increase coupling, particularly if a more proximal gap junction already existed between a pair of axons. Realistic coupling was achieved using around 100 gap junctions in the network of 30 dIN neurons. This implies, firstly, that many neuron pairs are coupled indirectly via the axon of a third. Secondly, each axon makes rather few gap junctions (5 to 8 gap junctions per axon). These numbers are similar to rat cerebellar basket cells where Alcami and Marty [64] used capacitative current measurements and paired recordings to estimate that each neuron was directly coupled to ~4 neighbours.

### Problems modelling small unmyelinated axons

Neurons with very fine, unmyelinated axons (< 0.5 μm in diameter) are widespread in the adult peripheral (pain pathways) and central nervous systems (e.g. hippocampal mossy fibres, parallel fibres of granule cells, olfactory receptor axons) but are also found in developing nervous systems like that of the tadpole. The function of such fine axons is not well understood because it is so difficult to obtain recordings from them [57,65,66]. The minimum diameter required to house their ‘molecular machinery’ is quite small but axons narrower than 0.1 μm may be prone to spontaneous initiation or failure of action potentials due to the stochastic nature of ion channel opening and closing [67,68]. In our modelling of the electrically coupled network of hindbrain dINs, axon diameter was found to be critical to achieving realistic coupling. Passive coupling between dINs could not be achieved with an axon diameter of less than 0.3 μm regardless of the gap junction layout and parameters. Measurements are not available for dINs but transmission electron microscope sections show that inhibitory neurons in the *Xenopus* tadpole have axon diameters of 0.2 to 0.9 μm with a mean of 0.4 μm [52]. The modelling therefore suggests there is a limit on minimum axon diameter if neurons are to be coupled electrically via their axons. However, we must remember that our model is not based on good data on the properties of fine unmyelinated axons which is just not available. Conclusions therefore have to be viewed with caution.

The effects of the densities and spatial distributions of membrane channels has been investigated in axons from many systems [57,69–72]. Modelling the population of tadpole dINs as a passive network suggested that most of them are coupled indirectly via the axons of other dINs. When the neurons were given active properties we found failures of action potential propagation over regions of axons with gap junctions; these failures were reduced if gap junction resistance or membrane excitability was increased. However, such failures are not compatible with physiological recordings which show that action potentials initiated in one dIN propagate reliably to cause synaptic potentials in more caudal neurons [45]. Simply increasing the density of sodium channels uniformly in the whole neuron or over the entire axon produced networks that were unstable and could fire repetitively even without stimulation. We have shown here how increasing the density of sodium channels and decreasing the density of potassium channels in the initial segment of the axon close to the soma radically increased the reliability of action potential propagation. Higher densities of sodium channels have been observed experimentally in the axon hillock and initial axon segment in other neuron types [57,69–72]. Action potential propagation failure near gap junctions may be reduced if there is localised clustering of sodium channels close to the gap junctions. Such clustering has been studied in models of invertebrate unmyelinated axons [73] and has been seen at the nodes of Ranvier in mammal myelinated axons [74]. Another possibility is the existence of two types of sodium channel: those near the gap junctions having a higher voltage activation threshold which allows them to conduct action potentials reliably, without initiating them spontaneously.

Finally, in other systems it is known that long duration action potentials may improve the reliability of action potential propagation in unmyelinated axons [75]. The duration of action potential in dINs is long compared to other neuron types in the tadpole [48]. Unfortunately, direct evidence on the membrane channels and currents underlying this long duration is not yet available [35,37,76] and a comparison of dIN recordings with our model dIN (Fig 6B) shows that model action potentials are shorter. Efforts were made to obtain a better match in our model dIN. Adding A-type potassium channels is already mentioned in the text. Increasing Ca channel density also lengthened the AP but led to depolarisation block when the dIN was depolarised. To avoid increased complexity which was not evidence based we accepted an action potential which is too short. Problems of action potential propagation may not be so significant in life if coupled neurons tend to receive similar input and fire synchronously, as is the case with tadpole dINs. This would reduce the voltage drop across the gap junctions and should make action potential propagation more reliable.

### Potential pitfalls with experimental techniques used to investigate electrical coupling and its significance

An unexpected outcome of modelling the tadpole dIN population was the demonstration that electrical coupling can have a misleading effect on neuron firing properties determined experimentally using whole-cell recordings. *In vivo*, in an unexcited network, step current injection into a single dIN always leads to a single action potential [45,48,77]. However, the modelling showed that electrical coupling can transform neurons that fire multiple action potentials in isolation so that they too only fire a single action potential at the onset of a step current injection (Fig 7). Our interpretation, based on results from the model, is that this restriction to a single spike when current is injected into a single dIN is caused by a combination of: 1) sodium channel inactivation and activation of a slow potassium channel; and 2) current flowing through the gap junctions which acts like a leak to counter depolarizing currents and prevent further firing. Both of these mechanisms are needed to prevent successive action potentials; individually they are not sufficient. We infer this because just prior to the first spike in a single depolarized dIN, currents will flow across gap junctions, but there is not yet any sodium inactivation or slow potassium channels activation and so the neuron is able to spike. On the other hand, when current is injected to depolarise all the dINs, current will not flow across gap junctions because the dINs will be roughly isopotential as they approach spike threshold, and the sodium inactivation and potassium currents are unable to prevent repetitive spiking (Fig 8). This would explain why individual dINs only ever fire a single spike in response to step current injection during whole-cell recordings. At present, it is impossible in experiments to inject current simultaneously into many dINs. However, it is possible to depolarise many dINs at the same time by perfusion of NMDA and this leads to repetitive firing [49] just as we have found with current injection into the whole model dIN population. These results illustrate how whole-cell recordings from electrically coupled neurons may give misleading evidence about their firing properties by significantly reducing their excitability [76].

Another experimental approach to the study of electrical coupling is the use of pharmacological blockers. In the model, removal of the coupling within a network increases individual dIN input resistances by ~50%. This is rather more than the 16 or 18% increases produced experimentally by gap junction blockers (flufenamic acid and 18-β-glycyrrhetinic acid respectively) despite their producing a "near-complete" block of electrical coupling [29]. This suggests that the action of the pharmacological blockers is not equivalent to the simple removal of gap junctions from the model, as would be the case for an ideal gap junction blocker. This reinforces the view that gap junction blockers are not specific and, as well as blocking electrical coupling, are likely to have more complex effects, such as on other membrane channels [21,28,78].

### Significance of electrical coupling and firing properties for rhythm generation

As in most rhythmic neuronal networks, two proposals have been made about the fundamental mechanisms producing rhythmic swimming-type activity in the tadpole. The first is a pacemaker mechanism [35,49,79]. It has been shown that when NMDA is applied to an isolated single side of the hindbrain, motor nerves and the dINs (which drive the swimming rhythm, [45,47]) fire rhythmically at frequencies similar to those seen during swimming, and that this does not depend on inhibition [49,80]. The evidence suggests that the excitatory dINs driving swimming have membrane properties which limit their repetitive firing, when they are excited, to within the normal swimming frequency range. The second proposal is a network mechanism [48,81,82] where reciprocal inhibitory synaptic connections between the two sides of the CNS play an important role in rhythm generation by producing PIR firing in dINs, when these are held depolarised by their own mutual NMDAR-mediated excitation. The most recent evidence suggests that the two mechanisms play complementary roles in generating a reliable and coordinated swimming rhythm [83] and ensuring that one side does not generate rhythmic motor activity on its own. In both mechanisms the dIN electrical coupling ensures that activity within each side in synchronised.

A long-standing paradox was that experimentally recorded dINs only fired a single spike to a depolarising current step (but see [76]**)**. They would therefore not be able to fire rhythmically when depolarised during swimming unless PIR allowed them to recover their excitability. However, our modelling shows that the restricted firing is a consequence of the electrical coupling, and that when the whole coupled dIN population is excited, the dINs are able to fire rhythmically as pacemakers. This suggests that PIR is not essential for rhythmic dIN firing during swimming, though it is still required to ensure antiphase coupling between the two sides.

In an electrically coupled network, the current flow between neurons at rest has the effect of reducing the excitability of the individual neurons. This has another possible significance for the response of tadpole dINs to synaptic input. The whole dIN population on one side of the CNS is normally recruited to fire together, following sensory stimulation of the skin, to elicit swimming [42]. We have shown in our model that electrical coupling can ensure step-wise whole-population recruitment. It could also work like the simple “coincidence detection” mechanism described for electrically coupled amacrine cells in the retina [9]. The result of the coupling would be that neurons are less likely to fire on their own and more likely to fire together in response to simultaneous synaptic excitation.

### Conclusions

Our modelling of axo-axonic gap junction coupling in the linear population of tadpole reticulospinal neurons generates a number of predictions that we expect to be relevant to other networks of coupled neurons with fine, unmyelinated axons. It predicts that axonal gap junctions will be located close to the neuron soma of the more caudal neuron and occur in low numbers (<8 hemichannels per neuron). Electrical coupling between neurons is therefore often indirect via the axons of other neurons. It may be possible to use tracers other than neurobiotin to locate gap junction contact points using dye filling of individual dINs to confirm that they are axo-axonic. It might also demonstrate indirect connections via other dINs. Neurobiotin injected with whole-cell electrodes does not appear to show dye coupling in the tadpole [29] although it has been used in other animals [84]. Our model showed that shunting through gap junctions can cause action potential propagation failure unless axonal membrane excitability is increased by changing the densities of sodium and potassium channels. In very small axons <0.3 μm in diameter propagation failure could not even be rescued in this way. The main, conventional role for electrical coupling in the tadpole reticulospinal population is to synchronize pacemaker firing during rhythmic activity and in this way coordinate motorneuron firing underlying swimming. However, electrical coupling also has dramatic effects on neuron firing properties measured experimentally using whole-cell recordings. Shunting can transform individual neurons able to fire repetitively into ones which only fire once to depolarising current when coupled. We plan to make voltage-clamp recordings to investigate the currents underlying the resting and active properties of dINs (cf. [37]). During such experiments it may be possible to physically isolate individual dINs by pulling them away with the whole-cell electrode and then testing if they fire repetitively to current injection when uncoupled from their neighbours. Our modelling emphasises how little is known about the physiology of fine unmyelinated axons in any part of any nervous system.

## Supporting Information

S1 Paper Simulations Contains the source code and instructions needed to run the simulations in this paper. (ZIP)
